# Hepatocyte growth factor regulates the TGF-β1-induced proliferation, differentiation and secretory function of cardiac fibroblasts

**DOI:** 10.3892/ijmm.2014.1782

**Published:** 2014-05-16

**Authors:** XIN YI, XIAOYAN LI, YANLI ZHOU, SHAN REN, WEIGUO WAN, GAOKE FENG, XUEJUN JIANG

**Affiliations:** Department of Cardiology, Renmin Hospital of Wuhan University and Cardiovascular Research Institute of Wuhan University, Wuhan, Hubei 430060, P.R. China

**Keywords:** hepatocyte growth factor, cardiac fibroblasts, fibrosis, c-Met, Akt

## Abstract

Cardiac fibroblast (CF) proliferation and transformation into myofibroblasts play important roles in cardiac fibrosis during pathological myocardial remodeling. In this study, we demonstrate that hepatocyte growth factor (HGF), an antifibrotic factor in the process of pulmonary, renal and liver fibrosis, is a negative regulator of cardiac fibroblast transformation in response to transforming growth factor-β_1_ (TGF-β_1_). HGF expression levels were significantly reduced in the CFs following treatment with 5 ng/ml TGF-β_1_ for 48 h. The overexpression of HGF suppressed the proliferation, transformation and the secretory function of the CFs following treatment with TGF-β_1_, as indicated by the attenuated expression levels of α-smooth muscle actin (α-SMA) and collagen I and III, whereas the knockdown of HGF had the opposite effect. Mechanistically, we identified that the phosphorylation of c-Met, Akt and total protein of TGIF was significantly inhibited by the knockdown of HGF, but was significantly enhanced by HGF overexpression. Collectively, these results indicate that HGF activates the c-Met-Akt-TGIF signaling pathway, inhibiting CF proliferation and transformation in response to TGF-β_1_ stimulation.

## Introduction

Heart failure, one of the major causes of mortality in industrialized countries, is a global chronic non-communicable disease of the 21st century (1). A recent study indicated that 5,800,000 Americans suffer from heart failure with a yearly incidence of 670,000 new cases and a yearly mortality rate of >280,000 individuals. Notably, more than one million hospitalizations occur each year, while the treatment cost is approximately 40 billion dollars (2). Heart failure is often accompanied by the occurrence of cardiac fibrosis, which can be initiated by various pathological factors, including hypertension, myocardial infarction and cardiomyopathy (3–5). Cardiac fibrosis is characterized by the pathological accumulation of extracellular matrix (ECM) mainly consisting of collagen (6–8). In the heart, collagen is primarily secreted by cardiac fibroblasts (CFs) (9). CF proliferation and transformation into myofibroblasts under pathological conditions play a key role in the process of cardiac remodeling (10–16). CF transformation into myofibroblasts is primarily promoted by transforming growth factor-β_1_ (TGF-β_1_), cytokines, the ECM and other growth factors, which result in the excessive production of ECM and the expression or secretion of growth factors; in the long term, the CFs become maladaptive and lead to abnormal myocardial stiffness, and ultimately, cardiac dysfunction (17,18). Therefore, the abrogation of CF transformation is one strategy for suppressing cardiac fibrotic remodeling that ultimately lead to heart failure.

Whereas many factors with a positive effect on CF activation have been described, relatively little is known about the factors that suppress the process of CF transformation (19–21). Of note, hepatocyte growth factor (HGF) is a potent antifibrotic cytokine that has been reported to have an antifibrotic function in various pathological conditions, such as pulmonary, liver and renal fibrosis (22–25). Previous studies have demonstrated that HGF inhibits α-smooth muscle actin (α-SMA)-positive myofibroblast activation from resident CFs induced by TGF-β_1_ or angiotensin II (26–28). These observations have led to the conclusion that HGF has great potential for negatively regulating CF function under pathological conditions, such as TGF-β_1_ stimuli. It is known that HGF has multiple biological functions through its specific tyrosine kinase receptor, c-Met (29,30). The binding of HGF to its receptor, an event that induces c-Met dimerization and activation, is then followed by the activation of multiple signaling pathways, such the ras-MAPK-ERK1/2, PI3K-Akt and TGF-Smad signaling pathways (31–33).

However, there is little information available as to how HGF affects the activation of CFs stimulated with TGF-β_1_. Therefore, the aim of the present study was to investigate the specific effects of HGF on the function of cultured CFs and to explore the mechanisms of action of HGF in CFs *in vitro*. In addition, we wished to determine whether HGF binds to its receptor, c-Met, and induces c-Met activation, subsequently activating related signaling pathways, e.g., the MAPK-ERK1/2, PI3K-Akt and TGF-Smad2/3 pathways.

## Materials and methods

### Materials

Primary antibodies against the following proteins were purchased from Santa Cruz Biotechnology, Inc. (Santa Cruz, CA, USA): HGF (sc-13087); TGIF (sc-9084); Ki67 (sc-7846); von Willebrand Factor (vWF) (sc-27649); antibodies against vimentin (ab8978), α-SMA (ab7817) were obtained from Abcam (Cambridge, MA, USA). Primary antibodies against MEK1/2 (no. 9122); ERK1/2 (no. 4695); phospho- MEK1/2^Ser217/221^ (Cat no. 9154); phospho-ERK1/2^Thr202/Thr204^ (no. 4370); phospho-Akt^Thr308^ (no. 13038); Akt (no. 9272); phospho-c-Met^Tyr1003^ (no. 3135); c-Met (no. 3127); phospho- Smad2^Ser465/467^ (no. 3101); Smad2 (no. 5339); phospho- Smad3^Ser423/425^ (no. 9520); Smad3 (no. 9523) and GAPDH (no. 2118) were from Cell Signaling Technology, Inc. (Beverly, MA, USA). Fetal bovine serum (FBS, no. SH30370.03) was purchased from HyClone (Logan, UT, USA). TRIzol was purchased from Invitrogen Life Technologies (Carlsbad, CA, USA) (no. 15596018). The BCA protein assay kit was purchased from Pierce Biotechnology, Inc. (Rockford, lL, USA) (no. 23235). Rat collagen I and III ELISA kits were obtained from NeoBioLab (Park Woburn, MA, USA) (no. RC0788 and no. RC0792). Cell culture reagents and all other reagents were purchased from Sigma (St. Louis, MO, USA).

### Cultured neonatal rat CFs and adenovirus infection

Primary cultures of neonatal rat CFs were prepared from the ventricles of 1- to 2-day-old Sprague-Dawley (SD) rats using the differential attachment method, as previously described (34,35). Briefly, the integrated hearts were removed and immediately placed in phosphate buffered saline (PBS), and the ventricles were minced, pooled, digested with 0.125% trypsin and 0.08% collagenase type II. The digestion was repeated 5 times. The collected cells were allowed to attach in DMEM/F12 medium supplemented with 10% FBS and incubated at 95% O_2_ + 5% CO_2_. After 1 h of attachment to the culture plates, the weakly attached or unattached cells were rinsed free and discarded, whereas the attached fibroblasts were grown in fresh DMEM/F12 supplemented with 10% FBS. Cells at passages 2–4 were used in the subsequent experiments. The identification of CFs was performed by immunofluorescence staining using anti-vWF for the detection of endothelial cells, anti-vimentin for fibroblasts and anti-α-SMA for cardiomyocytes. CFs at 80% confluence in the culture wells were digested by 0.25% trypsin and then passaged at 1:2 dilutions. The cells were stimulated with TGF-β_1_ with/without adenovirus transfection following starvation in serum-free DMEM/F12 for 12 h. AdHGF, expressing rat HGF recombinant adenovirus, was generated by subcloning full-length rat HGF cDNA downstream of the cytomegalovirus (CMV) promoter into a replication-defective adenoviral vector. A similar adenoviral vector encoding the *GFP* gene (AdGFP) was used as a control. Three silencing rat shHGF constructs were obtained from SABiosciences (Frederick, MD, USA) (KR44869H). Subsequently, AdshHGF adenoviruses were generated, and the construct that induced to the most significant decrease in HGF levels was selected for further experiments. AdshRNA was used as the control. The CFs were infected with recombinant adenoviruses at a multiplicity of infection (MOI) of 25 particles per cell for 24 h.

### Immunofluorescence staining

Immunofluorescence staining with the aforementioned antibodies was performed in the cultured CFs as previously described (36,37). Briefly, the CFs were washed 3 times with pre-cooling PBS, fixed with 4% paraformaldehyde and then permeabilized with 0.1% Triton X-100. Immunofluorescence staining was performed by incubating the CFs with vimentin, vWF, α-SMA and Ki67 primary antibodies overnight at 4°C. Following incubation with secondary antibodies for 60 min at room temperature, the CFs were incubated with DAPI for 10 min and mounted with aqueous mounting medium (Baso Diagnostics Inc., Taipei, Taiwan. Finally, the stained cells were visualized under a fluorescence microscope (Olympus Corp, Tokyo, Japan).

### Cell proliferation assay

Cell proliferation was assessed by cell counting kit-8 (CCK8) assay according to the manufacturer’s instructions. The cell suspension of CFs was inoculated into each 96-well plate at a density of 1×10^4^ cells/ml. After the CFs were treated with TGF-β_1_ with/without adenovirus infection, 10 μl of CCK8 solution were added to each well. The absorbance (A450) was measured to evaluate cell numbers and then estimate cell proliferation.

### Real-time polymerase chain reaction (PCR) analysis

Total RNA was extracted from the cells using TRIzol reagent according to the manufacturer’s instructions and the cDNA was synthesized using oligo(dT) primers with the transcriptor first-strand cDNA synthesis kit. Selected gene differences were confirmed by real-time PCR using SYBR-Green and the results were normalized against GAPDH gene expression. The sequences of all primers used in this study are presented in Table I.

### Western blot analysis

Total protein was extracted from the cultured CFs as previously described (38,39). Briefly, the lysates were collected by centrifugation at 10,000 × g. The protein concentrations were determined using a BCA protein assay kit. Fifty micrograms of protein extract were used for SDS-PAGE. The proteins were then transferred onto nitrocellulose membranes, blocked with 5% skimmed milk powder and then probed with various antibodies overnight at 4°C. Following incubation with a secondary peroxidase-conjugated antibody (Jackson ImmunoResearch Laboratories, Inc., West Grove, PA, USA; at a 1:10,000 dilution), signals were visualized with FluorChem E (Cell Biosciences, Santa Clara, CA, USA). Specific protein expression levels were normalized to GAPDH for total cell lysates and cytosolic proteins on the same nitrocellulose membrane.

### Enzyme-linked immunosorbent assay (ELISA)

Collagen I and III levels in the CF culture supernatant were measured using the the rat ELISA kits according to the manufacturer’s instructions. In brief, the culture supernatant was added to each ELISA plate well pre-coated with anti-rat collagen I and III polyclonal antibodies. Following 1 h of incubation at room temperature, the plates were washed and rat collagen I and III conjugate was then added to each well. The plates were incubated at room temperature for 2 h. The plates were then washed again, and substrate solution was added to each well. The plates were then incubated at room temperature in the dark for color development. After 30 min, stop solution was added to each well. The absorbance in each well was measured at 550 nm using a microplate reader. The concentrations of collagen I and III in the samples were determined by interpolation from the standard curve.

### Statistical analysis

The data are expressed as the means ± standard deviation (SD). Differences between various groups were tested with one-way ANOVA followed by the least significant difference (LSD) t-test using SPSS 13.0 statistical software. Comparisons between 2 groups were performed by an unpaired Student’s t-test. A value of P<0.05 was considered to indicate a statistically significant difference.

## Results

### Characterization of primary cultured neonatal rat CFs

**Figure 1:**
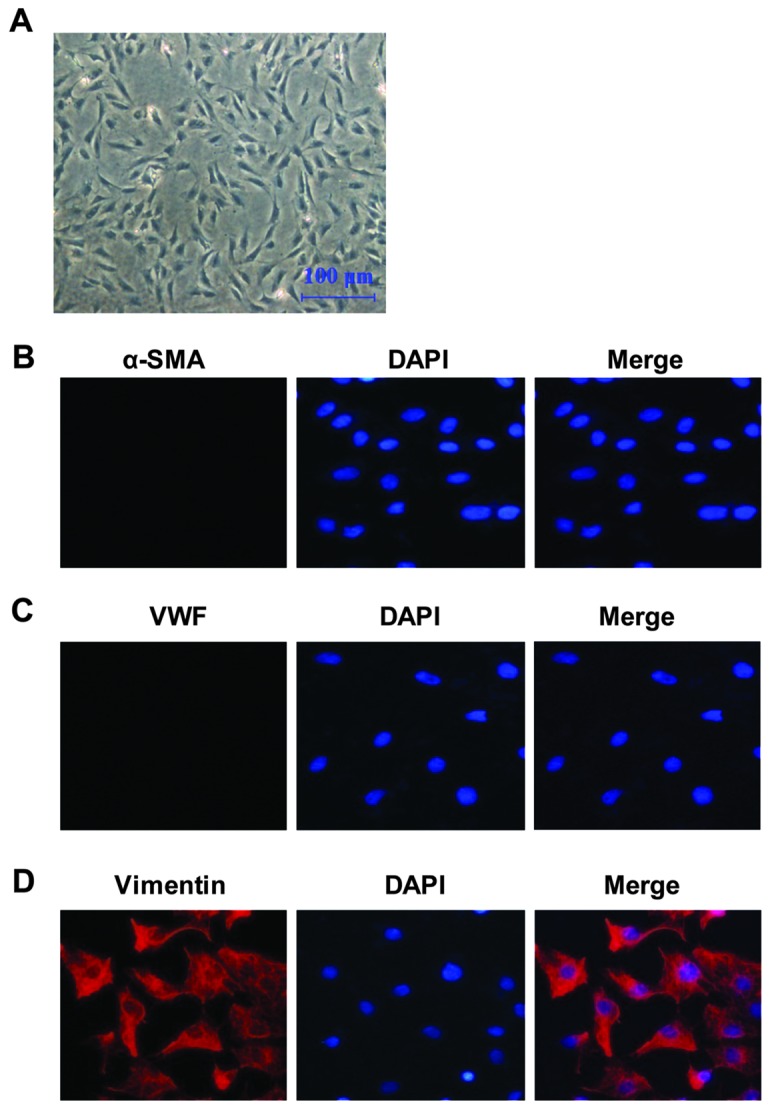
Characterization of primary cultured neonatal rat cardiac fibroblasts (CFs). (A) Cell morphology of first passage of neonatal rat CFs under an optical microscope (scale bar, 100 μm). (B–D) Primary cultured cardiac fibroblasts were subjected to immunofluorescence staining for (B) α-smooth muscle actin (α-SMA); (C) von Willebrand factor (vWF); and (D) vimentin to identify their purity [red, α-SMA/vWF/Vimentin staining; DAPI, blue (nuclear)]. Three independent experiments were performed.

The first passage of neonatal rat CFs cultured in our study had the typical morphological characteristics of fibroblasts, as evidenced by the spindle or polygonal shape of the cells, or the irregularly branched cytoplasm with a large ovoid nucleus (Fig. 1A) (40). The results of immunofluorescence staining revealed that these cells were negative for α-SMA and vWF, which are the markers of cardiomyocytes and endothelial cells, respectively (Fig. 1B and C) (41,42), but positive for vimentin, a marker of fibroblasts (Fig. 1D) (43). All these characteristics indicated that these cultured cells were CFs.

### HGF expression levels are decreased in CFs stimulated with TGF-β_1_

**Figure 2:**
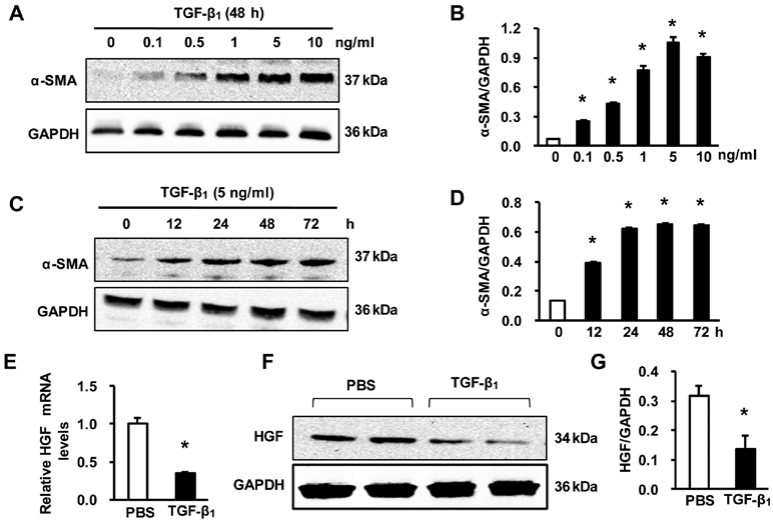
Hepatocyte growth factor (HGF) expression levels are decreased in cardiac fibroblasts (CFs) treated with transforming growth factor-β_1_ (TGF-β_1_). (A and B) The protein levels of α-SMA in primary cultured CFs treated with 0, 0.1, 0.5, 1, 5 and 10 ng/ml TGF-β_1_ for 48 h; (A) representative western blots; (B) quantitative results. ^*^P<0.05 vs. 0 ng/ml. (C and D) Protein levels of α-SMA in the samples from primary cultured CFs treated with 5 ng/ml TGF-β_1_ for 0, 12, 24, 48 and 72 h; (C) representative western blots; (D) quantitative results. ^*^P<0.05 vs. 0 h. (E) Relative mRNA levels of HGF in CFs following treatment with 5 ng/ml TGF-β_1_ for 48 h. ^*^P<0.05 vs. phosphate-buffered saline (PBS). (F and G) Protein levels of HGF in the samples from primary cultured CFs stimulated with 5 ng/ml TGF-β_1_ for 48 h; (F) representative western blots; (G) quantitative results. ^*^P<0.05 vs. PBS. Three independent experiments were performed.

To investigate the appropriate concentration and stimulation time of TGF-β_1_ in inducing CF transformation into myofibroblasts, we first measured the effects of several concentrations of TGF-β_1_ on inducing α-SMA protein expression after 48 h of incubation, which is the hallmark of myofibroblasts (44–46). It was found that the induction of α-SMA expression was highest when TGF-β_1_ was added at a concentration of 5 ng/ml (Fig. 2A and B). In addition, it was found that TGF-β_1_ induced α-SMA expression in the cultured CFs in a time-dependent manner. Compared with 12 and 24 h of incubation, the maximal expression of α-SMA protein was found at 48 and 72 h following stimulation with 5 ng/ml TGF-β_1_ (Fig. 2C and D) Therefore, we selected 5 ng/ml TGF-β_1_ with 48 h of incubation to induce CF transformation into myofibroblasts. To investigate the role of HGF in the function of CFs, we first measured HGF expression in the CFs that were stimulated with PBS or 5 ng/ml TGF-β_1_. Real-time PCR revealed that, compared with the PBS control, the mRNA levels of HGF were significantly downregulated in the fibroblasts stimulated with TGF-β_1_ for 48 h (Fig. 2E). This result was confirmed by western blot analysis, which showed that the HGF protein levels were reduced following treatment with 5 ng/ml TGF-β_1_ for 48 h (Fig. 2F and G). These results indicate that HGF may play a role in the function of CFs.

### Knockdown of HGF enhances the proliferation, transformation and secretory function of CFs

**Figure 3:**
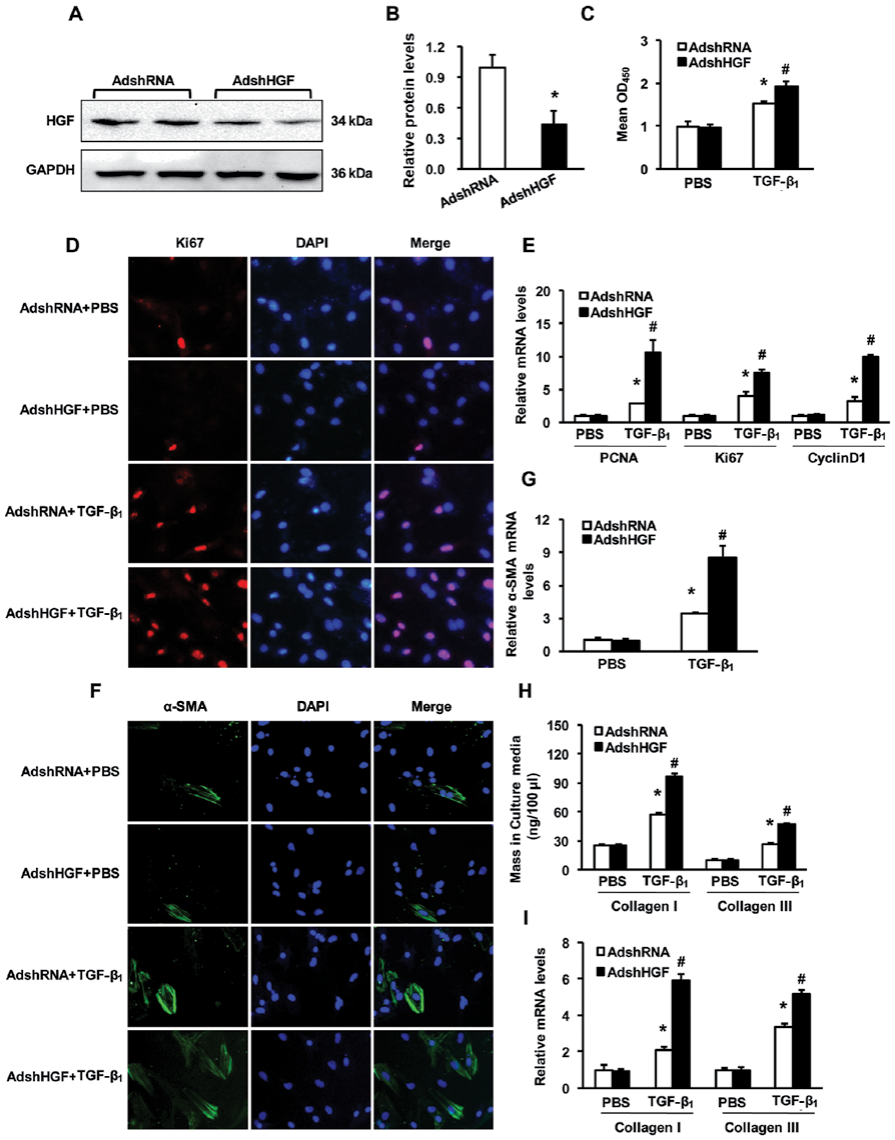
Knockdown of hepatocyte growth factor (HGF) in cardiac fibroblasts (CFs) enhances proliferation, transformation and secretory function. (A and B) HGF protein levels in CFs infected with AdshRNA or AdshHGF; (A) representative western blots; (B) quantitative results. ^*^P<0.05 vs. AdshRNA. (C) CCK8 assays were performed to measure the proliferation of CFs infected with AdshRNA or AdshHGF following stimulation with 5 ng/ml transforming growth factor-β_1_ (TGF-β_1_) for 48 h. (D) The immunostaining results showed that Ki67 expression levels was increased in AdshHGF infected CFs following stimulatin with 5 ng/ml TGF-β_1_ for 48 h compared with AdshRNA control (red, Ki67; blue, nuclear). (E) Relative mRNA levels of PCNA, Ki67 and cyclin D1 in samples from CFs of the indicated groups. (F) The α-SMA immunostaining in CFs of the indicated groups. [green, α-smooth muscle actin (α-SMA); blue, nuclear]. (G) Relative mRNA levels of α-SMA in CFs of the indicated groups. (H) Collagen I and III protein secretion in the culture medium of the indicated groups determined by enzyme-linked immunosorbent assay (ELISA). (I) Relative mRNA levels of collagen I and III in the CFs of the indicated groups. ^*^P<0.05 vs. AdshRNA/phosphate-buffered saline (PBS); ^#^P<0.05 vs. AdshRNA/TGF-β_1_. Three independent experiments were performed.

To define the functional contribution of HGF to the proliferation and transformation of CFs *in vitro*, the second passage of cultured neonatal rat CFs was infected with AdshHGF in order to knockdown HGF. As shown in Fig. 3A and B, the HGF protein levels were significantly reduced in the CFs infected with AdshHGF. Compared with the control (AdshRNA-infected), the CFs infected with AdshHGF presented an enhanced proliferation (Fig. 3C and D). Cell proliferation assay and the immunostaining of Ki67 revealed that TGF-β_1_ markedly enhanced the proliferation of the CFs compared with the PBS group, but this effect was further enhanced in the CFs in which HGF was knocked down (Fig. 3C and D). Additionally, we performed real-time PCR to determine the mRNA levels of proliferating cell nuclear antigen (PCNA), Ki67 and cyclin D1 in the CFs infected with AdshHGF/AdshRNA. The results revealed that, compared with the AdshRNA group, the mRNA levels of PCNA, Ki67 and cyclin D1 were markedly increased in the AdshHGF-infected CFs following stimulation with TGF-β_1_ (Fig. 3E). Under the appropriate conditions, resting or quiescent CFs can transform into myofibroblasts, which possess a more active, synthetic and contractile phenotype (47,48). We therefore determined whether HGF affects the phenotype switching of CFs following treatment with TGF-β_1_. The α-SMA levels in the CFs were determined by immunofluorescence and real-time PCR (Fig. 3F and G). Compared to the AdshRNA control group, the TGF-β_1_-induced expression of α-SMA was markedly increased by HGF knockdown (AdshHGF) (Fig. 3F and G). A number of studies have shown that, under inappropriate conditions, CFs may secrete fibrosis-related factors, such as collagen I and III, to promote the development of fibrosis (49–51). The results of ELISA showed that the collagen I and III levels were increased in the culture medium; this indicated that the AdshHGF-infected CFs had an enhanced secretory function (Fig. 3H). Moreover, the results of real-time PCR were consistent with the results of ELISA (Fig. 3I). Collectively, these loss-of-function data indicate that the knockdown of HGF enhances the proliferation, transformation and secretory function of CFs.

### Overexpression of HGF attenuates the TGF-β_1_-induced proliferation, transformation and secretory function of CFs

**Figure 4:**
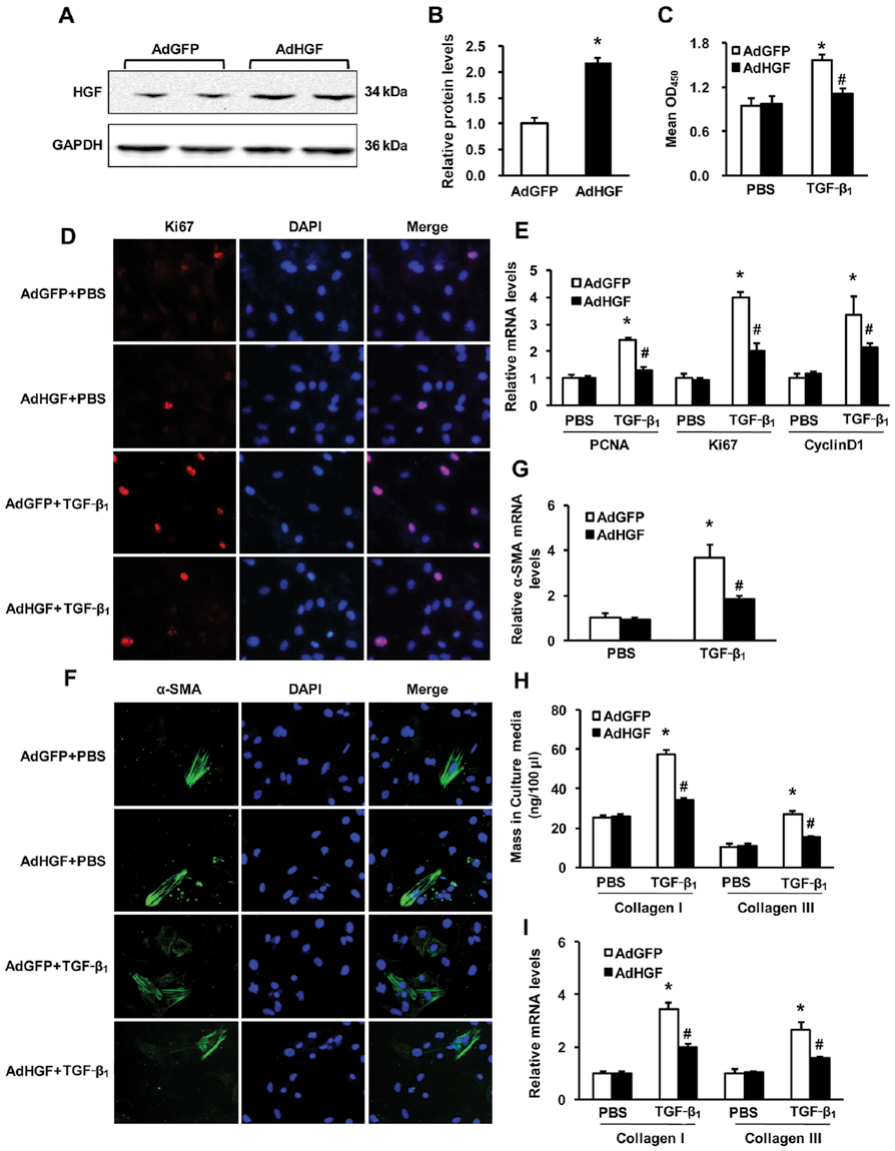
Overexpression of hepatocyte growth factor (HGF) in cardiac fibroblasts (CFs) attenuates transforming growth factor-β_1_ (TGF-β_1_)-induced proliferation, transformation and secretory function. (A and B) HGF protein levels in CFs infected with AdGFP or AdHGF; (A) representative western blots; (B) quantitative results. ^*^P<0.05 vs. AdGFP. (C) The CCK8 assays results indicated that the proliferation of CFs was suppressed by infection with AdHGF following stimulation with 5 ng/ml TGF-β_1_ for 48 h. (D) Repesentative images from immunofluorescence staining of Ki67 in CFs infected with AdGFP or AdHGF and stimulated with 5 ng/ml TGF-β_1_ for 48 h (red, Ki67; blue, nuclear). (E) The relative mRNA levels of PCNA, Ki67, and cyclin D1 in samples from CFs of the indicated groups. (F) Repesentative images from immunofluorescence staining of α-smooth muscle actin (α-SMA) in CFs infected with AdGFP or AdHGF and stimulated with 5 ng/ml TGF-β_1_ for 48 h (green, α-SMA; blue, nuclear). (G) Relative mRNA levels of α-SMA in CFs of the indicated groups. (H) Collagen I and III protein secretion in the culture medium of the indicated groups determined by ELISA. (I) Relative mRNA levels of collagen I and III in the CFs of the indicated groups. ^*^P<0.05 vs. AdGFP/phosphate-buffered saline (PBS); ^#^P<0.05 vs. AdGFP/TGF-β_1_. Three independent experiments were performed.

We then sought to examine whether increasing HGF levels in the CFs would attenuate their proliferation, transformation and secretory function. Thus, we overexpressed HGF in the second passage of cultured neonatal rat CFs by infection with AdHGF. The HGF protein levels markedly increased (~2.3-fold) in the CFs infected with AdHGF (Fig 4A and B). CF proliferation was then determined by CCK8 assay, and immunostaining of Ki67 and PCNA, Ki67 and cyclin D1 mRNA. Notably, AdHGF did not affect the proliferation of the CFs compared with the control AdGFP-infected cells treated with PBS. However, when the CFs were exposed to 5 ng/ml TGF-β_1_, the proliferation of the CFs was markedly inhibited by the overexpression of HGF compared with AdGFP (Fig. 4C–E). We also assessed the effects of HGF overexpression on the TGF-β_1_-induced transformation of CFs. The results of immunostaining and real-time PCR consistently showed that the α-SMA levels in the CFs were markedly reduced following stimulation with TGF-β_1_ compared with the AdGFP control (Fig. 4F and G). Moreover, ELISA was used to evaluate the levels of collagen I and III in the culture medium. The results indicated that the secretory function of the CFs was markedly inhibited by the overexpression of HGF following treatment with TGF-β_1_ (Fig. 4H); this was consistent with the results obtained by real-time PCR (Fig. 4I). Taken together, these data suggest that the overexpression of HGF suppresses the proliferation, transformation and secretory function of CFs.

### HGF activates c-Met-Akt-TGIF signaling, inhibiting the function of CFs

**Figure 5:**
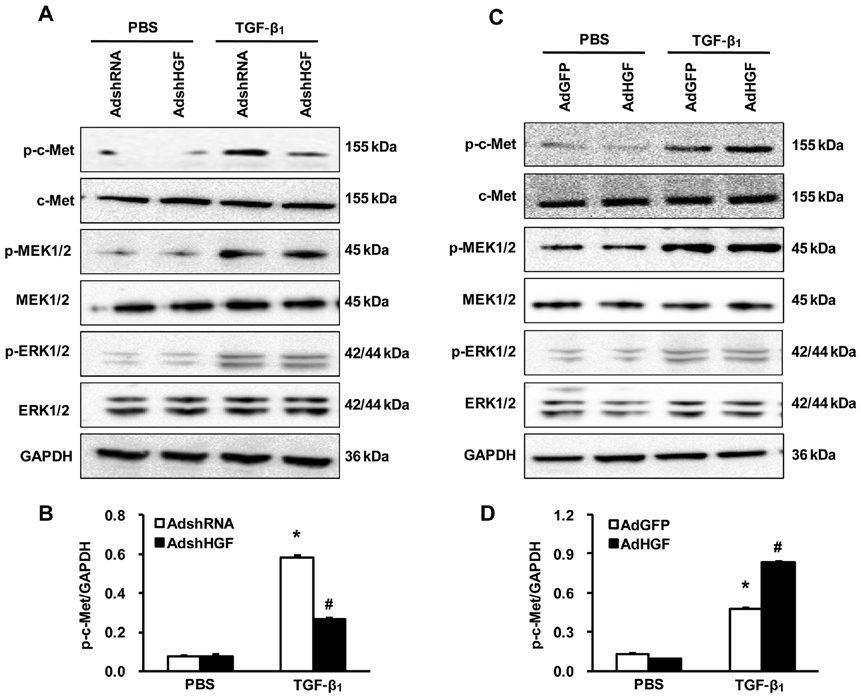
Effects of hepatocyte growth factor (HGF) on c-Met-MEK-ERK signaling. (A and B) Phosphorylation and total protein levels of c-Met, MEK1/2, ERK1/2 in AdshRNA- or AdshHGF-infected cardiac fibroblasts (CFs) stimulated with 5 ng/ml transforming growth factor-β_1_ (TGF-β_1_) for 48 h. (A) Representative western blots; (B) Quantitative results. (C and D) Phosphorylation and total protein levels of c-Met, MEK1/2 and ERK1/2 in samples from AdGFP- or AdHGF-infected CFs stimulated with 5 ng/ml TGF-β_1_ for 48 h. (C) Representative western blots; (D) quantitative results. Three independent experiments were performed. ^*^P<0.05 vs. AdshRNA or AdGFP/phosphate-buffered saline (PBS); ^#^P<0.05 vs. AdshRNA or AdGFP/TGF-β_1_.

As is known, HGF is the ligand of c-Met, and its phosphorylation activates downstream signaling (31–33). Thus, we first determined whether HGF affects the phosphorylation of c-Met following TGF-β_1_ stimulation. Western blot analysis with specific antibody to phospho-c-Met showed that the phosphorylation of c-Met was inhibited by the knockdown of HGF (AdshHGF), but was significantly enhanced by HGF overexpression (AdHGF) following TGF-β_1_ stimulation (Fig. 5). However, the phosphorylation of MEK1/2 and ERK1/2, downstream of c-Met, was not altered after the knockdown or the overexpression of HGF in the CFs stimulated with TGF-β_1_ (Fig. 5). These results indicate that HGF may not suppress the effects of TGF-β_1_ on CFs through the classic signaling pathway, MAPK-ERK1/2.

**Figure 6:**
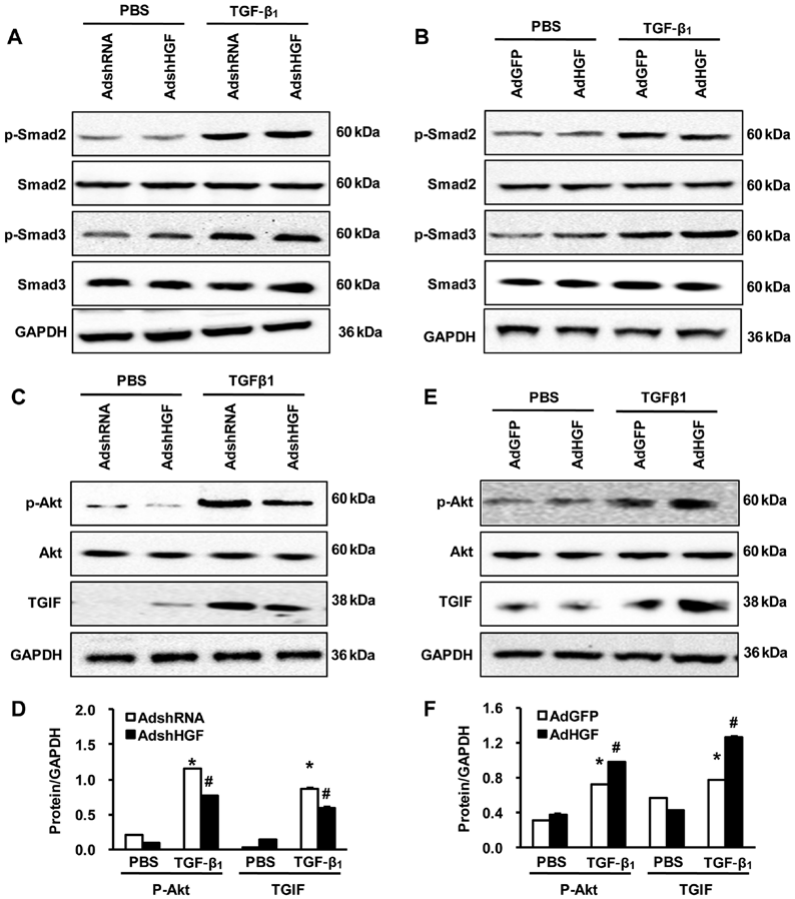
Effects of hepatocyte growth factor (HGF) on Akt-TGIF signaling. (A) Representative western blots showing the phosphorylation and total protein levels of Smad2 and Smad3 in AdshRNA or AdshHGF or (B) in AdGFP- or AdHGF-infected cardiac fibroblasts (CFs) stimulated with 5 ng/ml TGF-β_1_ for 48 h. (C–F) Phosphorylation and total protein levels of Akt and total protein of TGIF in AdshRNA- or AdshHGF-infected CFs stimulated with 5 ng/ml transforming growth factor-β_1_ (TGF-β_1_) for 48 h. (C and E) Representative western blots; (D and F) quantitative results. Three independent experiments were performed. ^*^P<0.05 vs. AdshRNA or AdGFP/phosphate-buffered saline (PBS); ^#^P<0.05 vs. AdshRNA or AdGFP/TGF-β_1_.

In order to gain insight into the molecular mechanisms underlying the negative role of HGF in the function of CFs we observed *in vitro*, we investigated whether the phosphorylation of Smad2 and Smad3 is enhanced in Cfs infected with AdshRNA and AdGFP following stimulation with TGF-β_1_. We found that there was no difference between the CFs infected with AdshRNA/AdshHGF or the CFs infected with AdGFP/AdHGF following treatment with TGF-β_1_ (Fig. 6A and B). Notably, the phosphorylation of Akt and total protein of TGIF (a Smad transcriptional co-repressor) was significantly suppressed in the CFs in which HGF was knocked down, but increased in the CFs in which HGF was overexpressed following TGF-β_1_ stimulation (Fig. 6C–F). Collectively, these data indicate that HGF positively regulates the c-Met-Akt-TGIF signaling pathway, inhibiting the function of CFs in response to TGF-β_1_ stimuli.

## Discussion

In the present study, we investigated the effects of HGF on the response of cultured CFs, as well as the possible mechanisms of involved. CFs were important in the cardiac remodeling process. We demonstrated that: i) TGF-β_1_ induced α-SMA expression in the cultured CFs in a time- and concentration-dependent manner; the maximal expression of α-SMA protein was investigated in the CFs treated with 5 ng/ml TGF-β_1_ for 48 h; ii) HGF inhibited the proliferation of CFs; iii) HGF inhibited the differentiation of CFs into myofibroblasts; iv) HGF decreased the synthesis and secretion of collagen I and III in the CFs; v) HGF activated the phosphorylation of its receptor, c-Met; vi) TGF-β_1_ stimulation increased the phosphorylation of MEK1/2 and ERK1/2, downstream of c-Met, but the knockdown or the overexpression of HGF did not alter the phosphorylation levels of the aforementioned signaling molecules; vii) the phosphorylation of Smad2 and Smad3 was enhanced in the CFs following stimulation with TGF-β_1_, but there was no difference between HGF knockdown and HGF overexpression; viii) the phosphorylation of Akt and total protein of TGIF were significantly suppressed in the CFs in which HGF was knocked down, but increased in the CFs in which HGF was overexpressed following TGF-β_1_ stimulation.

CFs represent the largest class of cells residing in the normal heart, and the proliferation of CFs is the main characteristic of cardiac fibrosis (52,53). In the present study, by CCK8 assay, we investigated whether HGF inhibits CF proliferation, as indicated by the decreased mRNA levels of CF proliferation markers, such as Ki67, PCAN and cyclin D1. Hence, HGF is effective for the treatment of cardiac fibrosis. The phenotypic transformation of CFs into myofibroblasts is known to be another key event in the process of cardiac remodeling. Under abnormal conditions, the persistence of myofibroblasts can facilitate hypertrophy and fibrosis, which results in structural remodeling and cardiac dysfunction (54,55). Therefore, the prevention of myofibroblast transformation may be a potential therapy aiming at limiting cardiac fibrosis. Cardiac myofibroblasts are greatly active cells that express α-SMA and exhibit increased synthesis and secretion of massive collagen proteins, such as collagen I and III (56–58). In this study, we demonstrated that HGF significantly attenuated the α-SMA and collagen I and III expression levels in the CFs. The present study using cultured CFs confirmed the beneficial effects of HGF shown in animal models (59–61). However, the most impressive finding of the present study was that HGF positively inhibited the function of CFs in response to TGF-β_1_ stimuli by regulating the c-Met-Akt-TGIF signaling. As is known, HGF is the ligand of c-Met, and its phosphorylation activates downstream signaling (31–33). Thus, we first determined that the phosphorylation of c-Met was inhibited by the knockdown of HGF, but was significantly enhanced by HGF overexpression following TGF-β_1_ stimulation. Furthermore, the downstream signaling of c-Met and the phosphorylation of Akt were significantly suppressed follwoing HGF knockdown, but increased following HGF overexpression. However, the phosphorylation of MEK1/2 and ERK1/2, classic downstream signaling molecules of c-Met, was not altered after the knockdown or overexpression of HGF in the CFs stimulated with TGF-β_1_, indicating that HGF may not restrain the function of TGF-β_1_ on CFs through the classic MAPK-ERK1/2 signaling pathway.

Smad proteins are thought to play an important role in regulating intracellular responses to TGF-β_1_. Smad proteins, such as Smad2 and Smad3, are activated by TGF-β_1_ receptors and then translocate to the nucleus, where they regulate transcription, further modifying multiple CF functions, including proliferation, differentiation and secretion. Indeed, our study demonstrated that TGF-β_1_ induced Smad2 and Smad phosphorylation in the CFs. However, HGF failed to attenuate TGF-β_1_-induced Smad2 and Smad3 phosphorylation. Notably, the protein levels of TGIF, a Smad transcriptional co-repressor (62), were significantly suppressed in the CFs in which HGF was knocked down, but increased in the CFs in which HGF was overexpressed following TGF-β_1_ stimulation. These findings indicate that in CFs, HGF blocks the TGF-β_1_-induced nuclear translocation of phospho-Smad2 and phospho-Smad3, inhibiting CF proliferation, differentiation and secretion. Similarly, HGF suppresses TGF-β_1_-mediated renal interstitial myofibroblast activation, and this effect of HGF is likely related to the blockade of Smad nuclear translocation (63).

In conclusion, the results of the present study suggest that HGF exerts its antifibrotic effects on CFs by inhibiting CF proliferation and transformation. The underlying mechanism is that HGF positively regulates c-Met-Akt-TGIF signaling to inhibit the function of CFs in response to TGF-β_1_ stimuli. However, a limitation of this study was that the detailed mechanism by which HGF regulates cardiac fibrosis remains to be elucidated using animal models with TGF-β_1_ stimuli. Another limitation was that even though we found that the protein expression of TGIF was significantly affected by HGF, other Smad transcriptional co-repressors, such as c-Ski, Ski-related novel protein N (SnoN), ecotropic virus integration site 1 protein homolog (Evi1), Smad interacting protein 1 (SIP1) and histone deacetylase (HDAC)4/5, remain to be explored.

## Figures and Tables

**Table I:** Primer pair sequences used for the real-time PCR analysis of gene expression.

Gene	Forward	Reverse
GAPDH	GGGTGATGCTGGTGCTGAGTATGT	CAGTGGATGCAGGGATGATGTTCT
PCNA	CAACTTGGAATCCCAGAACAGGAG	TAAGGTCCCGGCATATACGTGC
Ki67	TAGAGGATCTGCCTGGCTTC	TGTCCTTGGTTGGTTCCTCC
α-SMA	GCTCTGTAAGGCGGGCTTTG	ACGAAGGAATAGCCACGCTCA
Cyclin D1	GAACTACCTGGACCGTTTCTTG	AGGAAGTGTTCGATGAAATCGT
Collagen I	GAGCGGAGAGTACTGGATCGA	CTGACCTGTCTCCATGTTGCA
Collagen III	TGCCATTGCTGGAGTTGGA	GAAGACATGATCTCCTCAGTGTTGA
HGF	ATCAGACACCACACCGGCACAAAT	GAAATAGGGCAATAATCCCAAGGAA

PCR, polymerase chain reaction; PCNA, proliferating cell nuclear antigen; α-SMA, α-smooth muscle actin; HGF, hepatocyte growth factor.
